# Clinical and Pathological Features of Angiomatous Nasal Polyps: A Report of Four Cases and Review of Literature

**DOI:** 10.7759/cureus.7642

**Published:** 2020-04-11

**Authors:** Kholood S Assiri, Mohammad S Al-Ahmari, Mohammad S Alshahrani, Ali Mastor, Reda Elhawary

**Affiliations:** 1 Otorhinolaryngology, King Faisal Medical City, Abha, SAU; 2 Otolaryngology, Khamis Mushayt General Hospital, Khamis Mushayt , SAU; 3 Otolaryngology, Khamis Mushayt General Hospital, Khamis Mushayt, SAU; 4 Pathology, Al-Azhar University, Cairo, EGY; 5 Pathology, Khamis Mushayt General Hospital, Khamis Mushayt, SAU

**Keywords:** angiomatous nasal polyp (anp), diagnosis, vascular proliferation, endoscopic sinus surgery

## Abstract

Inflammatory or allergic sinonasal polyps are characterized by extensive vascular growth and ectasia with deposition of pseudoamyloid in 5% of cases. Angiomatous nasal polyp (ANP) is a relatively rare benign lesion, which may be misdiagnosed as a benign or malignant tumor. The characteristic pathological features of ANP are expanded angiogenesis, accumulation of extracellular amorphous eosinophilic substance, and atypical stromal cells. This report aimed to outline the histological differential diagnosis of ANP.

Through a full histopathological examination, we studied biopsies and resected specimens from five patients who were diagnosed with ANPs, including one with facial deformity. Gross findings showed that tumors were firm in consistency, lobulated on the surface, and lined by partially ulcerated mucous membrane. Light microscopy showed clusters of widened, thin-walled blood vessels among congo red-negative eosinophilic substance with an area of necrosis and irregular stromal spindle cells. Presence of endothelial cell myofibroblasts were confirmed by electron microscopy and immunohistochemistry.

This is a report of four cases which showed extreme examples of ANPs that was completely resected by endoscopic sinus surgery for all patients. A full histopathological examination is recommended to confirm the possible differential diagnoses for a better management plan.

## Introduction

Inflammatory sinonasal polyps are assorted into main five variants: edematous, glandular, fibrous, cystic and angiomatous polyp. Clinically, patients may experience a gradual obstruction of the nasal cavity, loss of smell sensation, nasal discharge or epistaxis. Also, the mass could cause exophthalmoses, and visual disturbances. Angiomatous nasal polyps (ANPs) are relatively rare benign lesions recognized by extensive vascular growth and ectasia, with scant of inflammatory cells and abundant extracellular fibrin [1-4]. Clinically, patients may experience a gradual obstruction of the nasal cavity, loss of smell sensation, nasal discharge or epistaxis. Also, the mass could cause exophthalmoses, proptosis and visual disturbances [3].

Furthermore, inflammatory nasal polyps could be misdiagnosed histologically with other tumors including papilloma, squamous cell carcinoma, nasal lymphoma, and other soft tissue neoplasms in the nasopharynx [5, 6]. Few reports have concluded that sinonasal polyps with profuse vascularity are easily confused with multiple vascular tumors such as nasopharyngeal angiofibromas [7,8]. However, the microscopic features for ANPs have not been addressed well enough in several studies [9, 10, 11, 12, 13]. 

In this article, we aim to represent clinical, radiological and pathological features of ANP of the paranasal sinuses that resulted in bone erosion and expanded to the right nasal cavity and the ipsilateral pterygopalatine fossa. 

## Case presentation

Case 1

A 53 years old female with known history of hypertension presented with a chief complaint of left progressive nasal obstruction and nasal discharge for a duration of six months. She had no aggravating or alleviating factors and had no otological symptoms (ear pain, deafness, sensation of fullness, tinnitus, and dizziness), postnasal drainage, epistaxis, facial numbness, diplopia and any impairment in her visual acuity.

**Figure 1:**
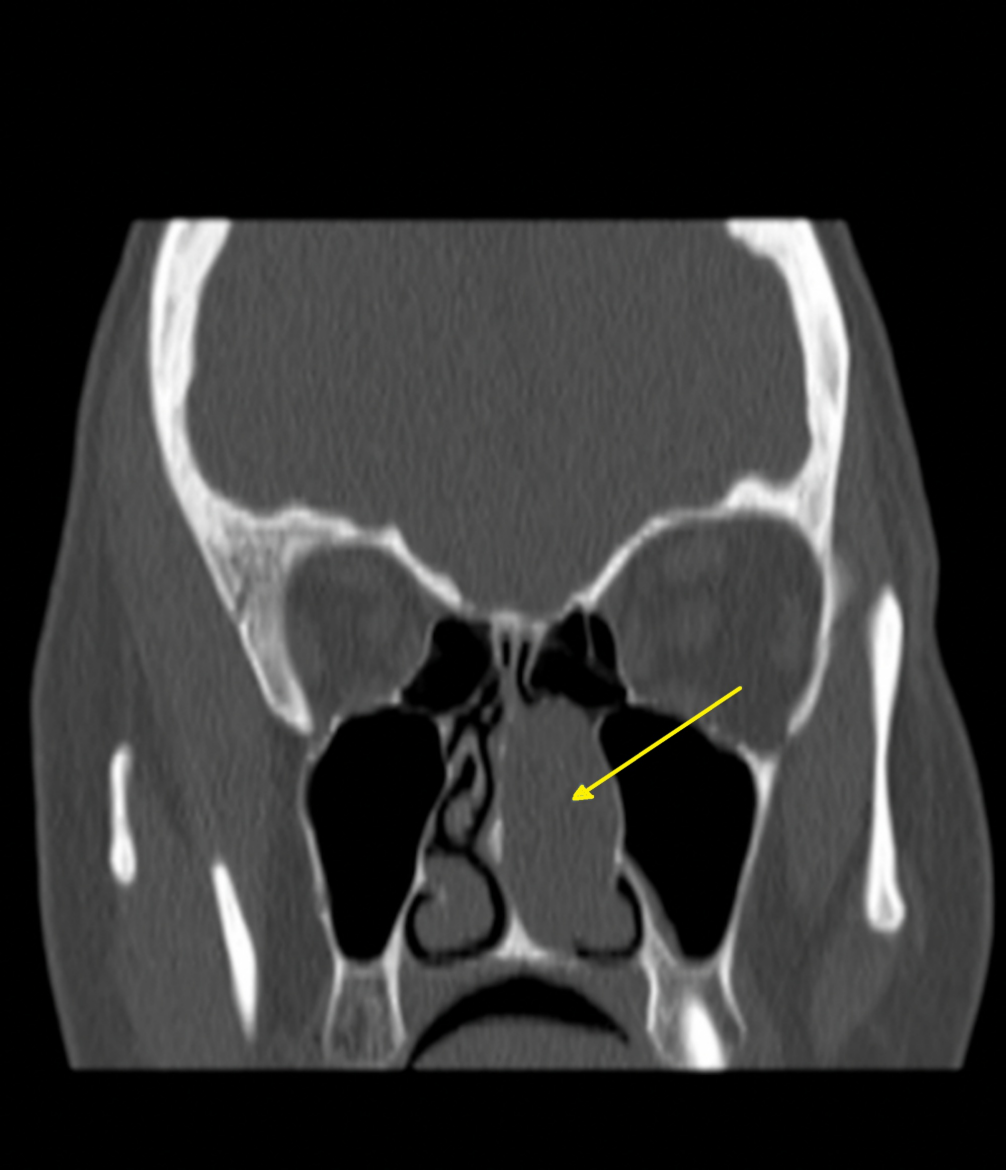
Shows lesion occupying left nasal cavity

On clinical examination, the patient was generally stable. However, she had a grade two left nasal polyp, a (R/L) deviated nasal septum, and normal cranial nerve exam. There were no other remarkable findings on her ENT examinations. Her laboratory investigations were within normal limits. A CT was performed which showed left homogenous soft tissue mass in the nasal cavity extending to the left maxillary ostium (Figure 1). Septoplasty and removal of leasion done through Endoscopic Sinus Surgery were performed and the polyp was sent for histopathological evaluation. Follow-up at 4 months, 8 months , and 1 year revealed entire improvement of her complaints. 

Case 2

A 31-year-old female presented with bilateral nasal obstruction and nasal discharge for more than one year. These symptoms were progressive in their course and without aggravating and relieving factors were present. This was associated with headache and anosmia with no history of epistaxis, visual loss, diplopia or otological symptoms. The patient reported no fever, weight loss or change in appetite. The patient underwent functional endoscopic sinus surgery for bilateral anterior and posterior ethmoidectomy, bilateral maxillary antrostomy and bilateral sphenoidotomy seven years ago. Biopsy was obtained with no obvious histopathological findings.

**Figure 2:**
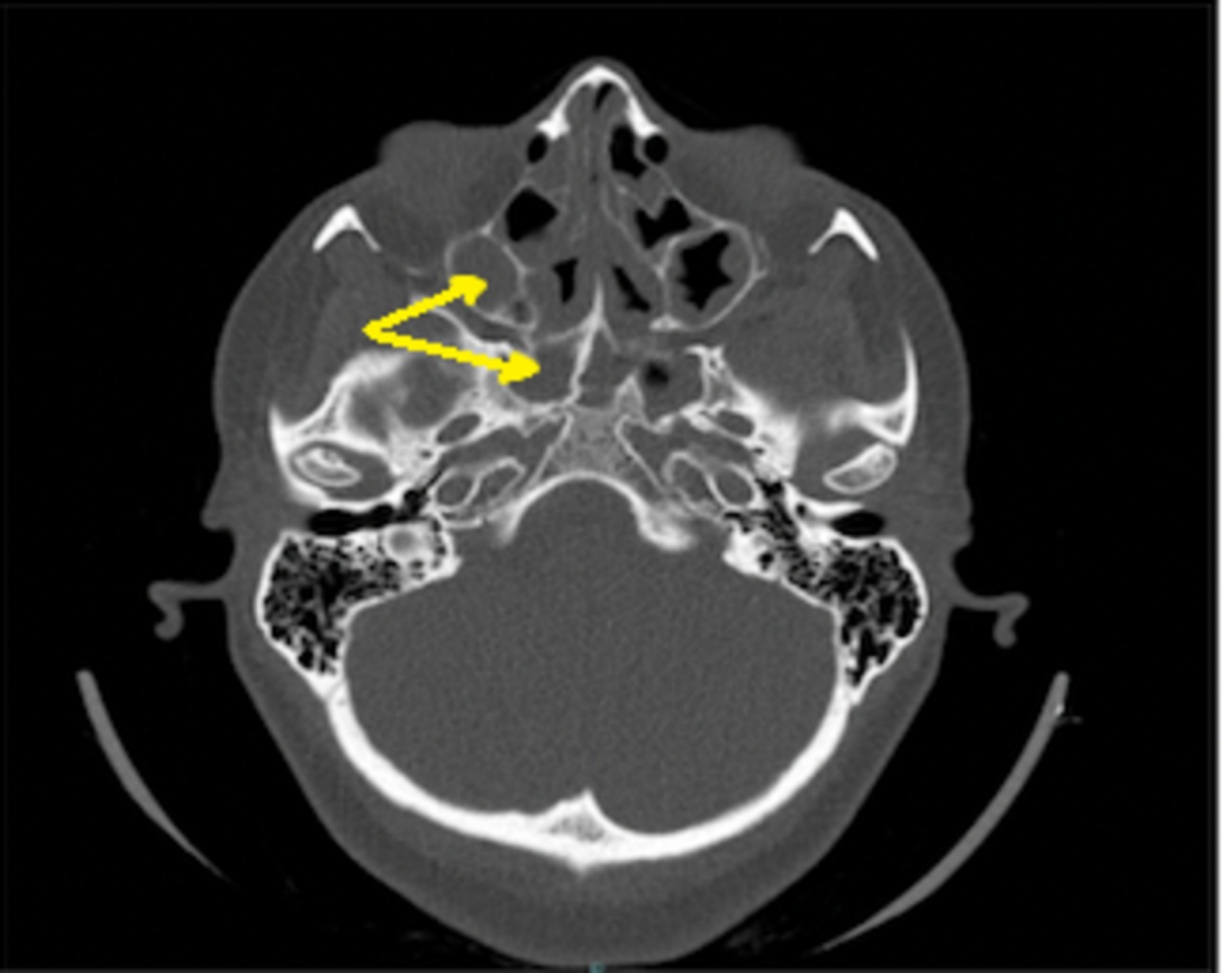
Shows recurrent sinonasal polyposis

On clinical examination, the patient was conscious, oriented, and hemodynamically stable. By local examination, bilateral nasal polyp and hypertrophy inferior turbinate were noted. No visual loss or diplopia was present. Remaining ENT examinations were unremarkable. Her laboratory investigations were within normal limits. A CT scan showed opacification of all paranasal sinuses, without any bony erosion. Endoscopic Sinus Surgery was performed and polyp was sent for histopathology department. Follow up of this case after five years shows disease recurrence (Figure 2).

Case 3

**Figure 3:**
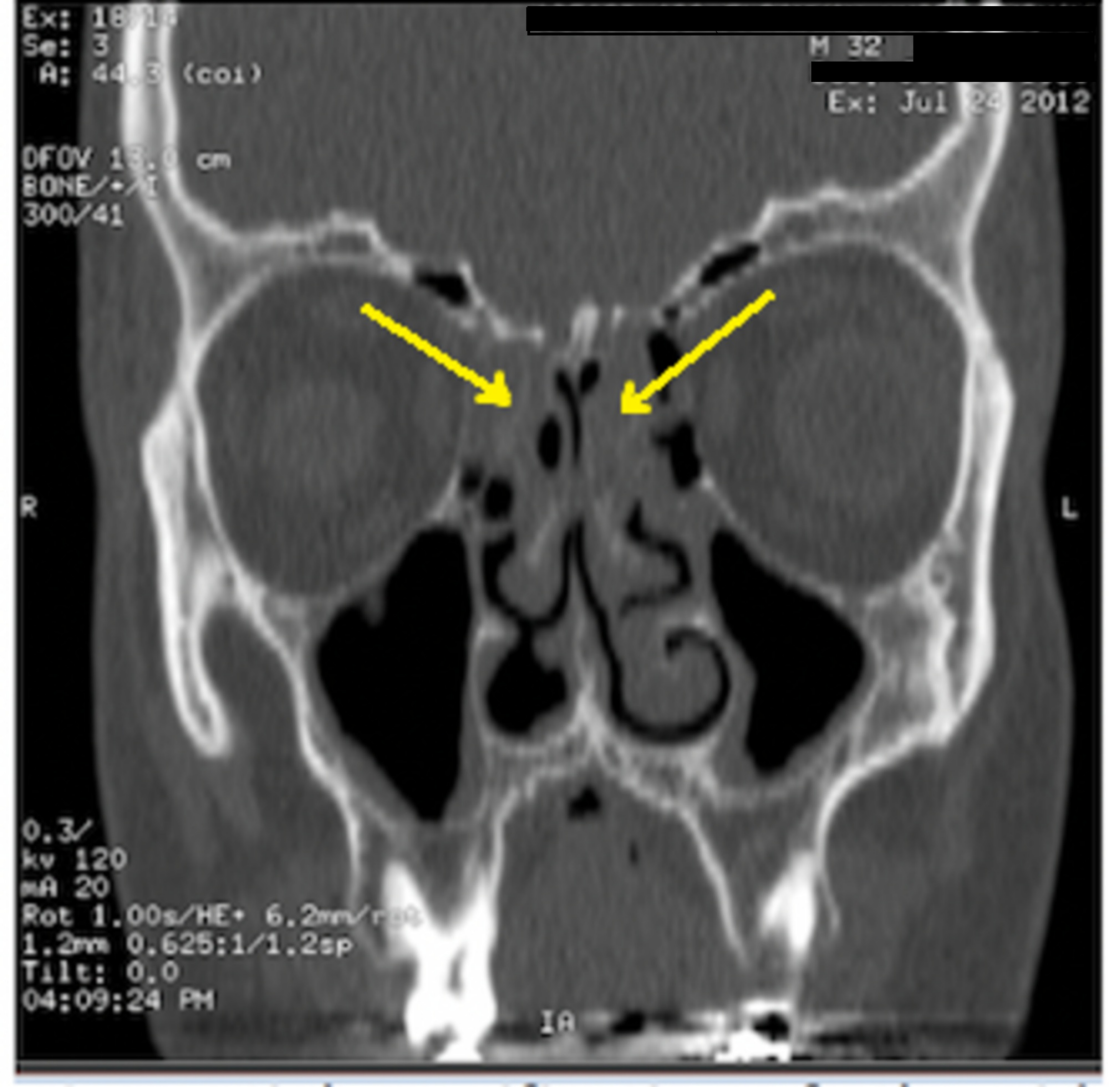
Shows bilateral partial opacification of ethmoid sinuses

A 32-year-old male came with a chief complaint of bilateral nasal obstruction and nasal discharge for more than one year; these symptoms were progressive. No aggravating or alleviating factors were present. The condition was associated with headache. The patient reported no history of epistaxis, visual loss, diplopia or otological symptoms. However, the patient was known to have a history of allergy to dust. Two years ago, the patient had similar surgical intervention as case 2, and also with no obvious histopathological finding. On examination, bilateral nasal polyp were found. No visual loss or diplopia was reported and other ENT examinations were unremarkable. His laboratory investigations including full blood picture, coagulation profile, kidney function test, and hepatic panel were within normal limits. A CT scan showed partial opacification of ethmoid sinuses without any bony erosion (Figure 3). Endoscopic Sinus Surgery was performed and the entire lesion was excised and sent for histopathological examination.

Case 4

A 14-year-old male came with sudden onset left facial swelling. He noted two months of progressive left nasal obstruction, nasal discharge and headache. The patient had no history of epistaxis, otological symptoms, visual loss or diplopia. However, there was a history of allergy to dust. On the clinical examination, the patient was alert, oriented, and vitally stable. By the local examination, left facial swelling was observed. Endoscopy revealed a left nasal friable mass with no bleeding. No visual loss or diplopia was noted. Other ENT examinations were unremarkable.

His laboratory investigations (as in the previous case) were within normal limits. A CT scan indicated a soft tissue mass in the left nasal cavity showing marked hypervascularity expanding the left maxillary ostium, extending anteriorly to the left check, and to the posterior naris on the left side including the left side of pterygoplatine fossa.

Under general anesthesia, a biopsy was taken and sent as frozen section because of a high suspicion of malignancy. After the result of histopathology, the lesion was excised without any complications and had no index of recurrence throughout the follow-up time. In the operation, the tumor was restricted in the left nasal cavity and reached about 6 cm in diameter with a little pedicle that emerged via the middle meatus.

Results of Light Microscopy 

**Figure 4:**
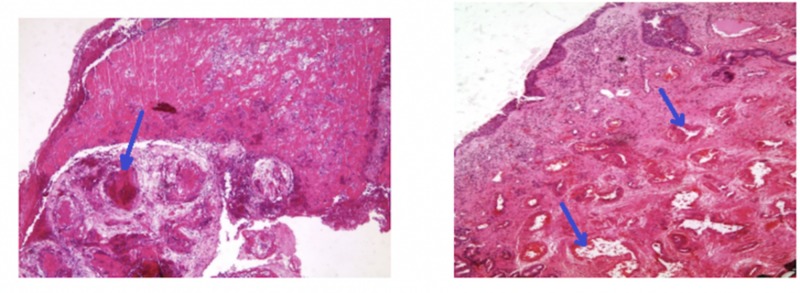
Variable sized small and ectatic thin walled blood vessles , with foci of vascular congestion.

**Figure 5:**
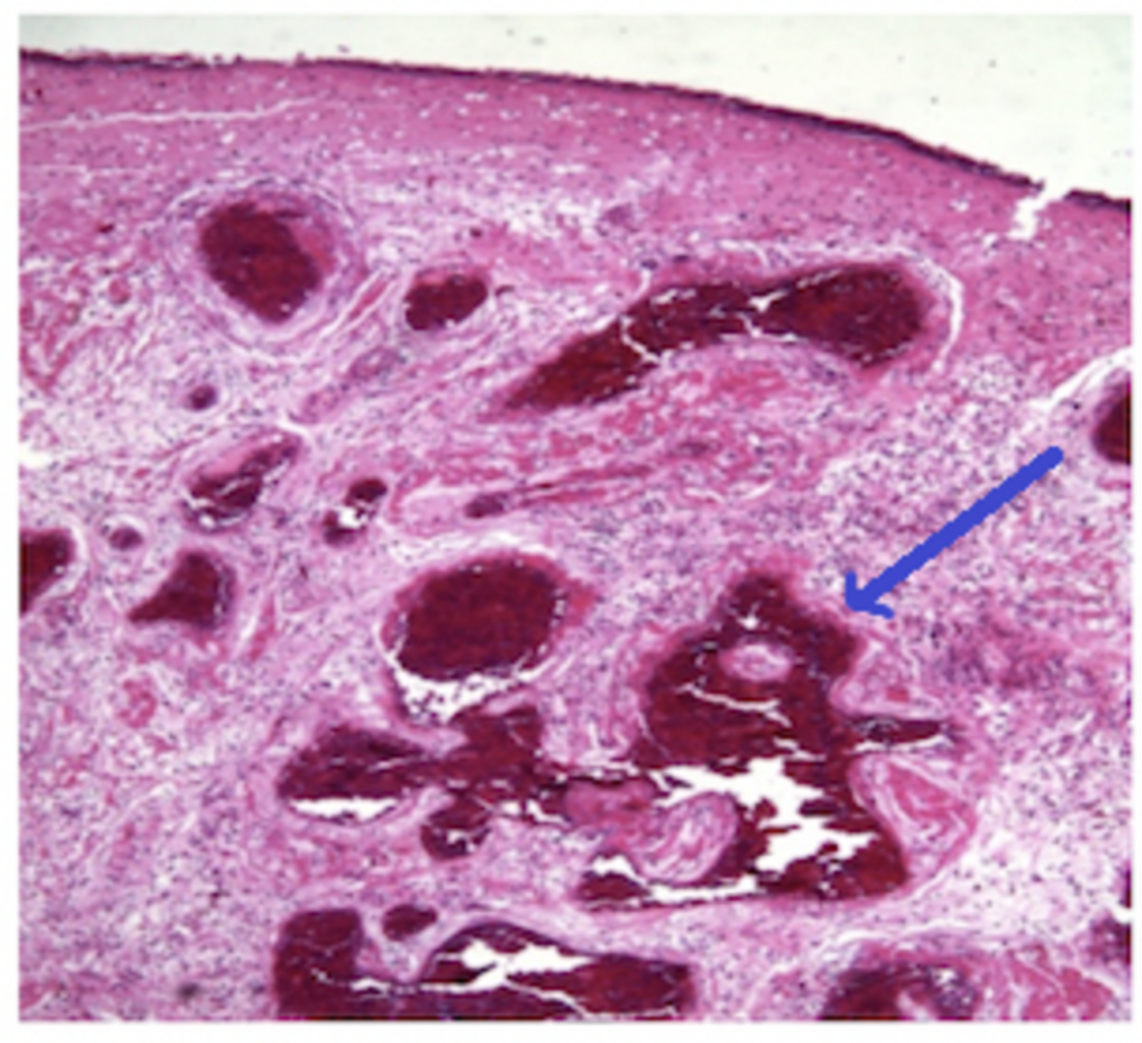
Angiectatic polyps with vascular thrombosis

The roofs of the lesions were ulcerated with areas lined by pseudostratified ciliated respiratory epithelium with squamous metaplasia. Thin-walled vessels were detected in almost all cases; they were irregular in shape and encircled by extensive Congo red negative eosinophilic extracellular material. Fibrin thrombi are also noted in some blood vessels lumina. The stroma was dispersed with irregular spindle cells, and the vascular spaces were covered by endothelial cells (Figure 4). The atypical stromal cells were enlarged with vesicular nuclei, eminent nucleoli, and eosinophilic cytoplasm. There were alternating areas of cavernous-type blood vessels and vascular zones. Cystic degeneration was also noted inside pseudopapillary projections lined by endothelium. In case 4, typical angiectatic nasal polyps and vascular thrombosis were demonstrated (Figure 5).

Immunohistochemical Studies

Endothelial cells were demonstrated in the vascular-looking channels and papillary-like projections through immunoperoxidase stains for endothelial markers (factor VIII, and CD34). Further, desmin and smooth muscle actin were positively stained in the normal sized walls and the cytoplasm of rare atypical and non-atypical stromal cells. However, in the widened and cavernous vascular spaces, they were negatively stained. In addition, cytokeratin stains were positive in the epithelial part only.

## Discussion

ANPs are deemed infrequent lesions, representing only 4-5% of whole nasal polyps [13]. Batsakis et al. reported that angiectatic nasal polyps are deemed types of antrochoanal polyp [2, 14]. They postulated that vascular pressure of the polyp at the ostium causes necrosis and then reparative changes and angiogenesis [14].

In angiectatic polyps, presence of scattered atypical pleomorphic spindle cells (Myofibroblasts) in the stroma is frequent; however, they are less frequent in sinonasal polyp [4, 15]. In addition, most of detected non-epithelial tumors of the nasal cavity and nasopharynx are vascular types [12]. Thus, the report by Wang YZ et al. declared that the diagnosis of ANPs is challenging with several differentials such as with capillary or cavernous hemangioma and nasopharyngeal angiofibroma [16,17].

Majority of the ANPs originate in the maxillary sinus and expand across the choana and into the nasopharynx [3, 16] Progressive expansion of the ANPs may lead to erosion and disruption of the surrounding bony parts, swollen face, and proptosis [18]. Although there are no established guidelines for management, the transnasal endoscopic removal is considered the best therapeutic option of choice since it has good prognosis and low level of recurrence [19].

On the other hand, Yfantis et al. mentioned that in certain cases, angiomatous nasal polyps may lead to massive bone erosion and/or nasal bleeding. The incidence of any of the aforementioned symptoms may consolidate the clinical doubt of the presence of malignant lesion [4]. Generally, angiomatous nasal polyps have a chronic disease history. Angiomas are consisted of atypical vascular tubes with padded flat endothelial cells incorporated in edematous stroma. Sinonasal angiomas are detected commonly in anterior nasal septum, vestibule, or concha, and manifest with nasal blockage and bleeding without gender or age difference [12].

Dai et al. and Yfantis et al. observed that the most noteworthy pathological characteristics of ANP were the great quantity of blood vessels, evidence of intravascular thrombosis, the massive necrosis of the lesion, and the extravasation of the blood composition into the nearby stroma [2, 4]. The features of ANP under light microscopy are as follows: (i) racemose aggregates of irregularly shaped blood vessels resembling dilated capillaries and no elastic or muscular layers (ii) acute and chronic inflammation common, hemosiderin-laden macrophages (iii) heterogeneity from field to field and patchy areas with features of typical inflammatory polyps; (iv) paucicellular stroma with scattered fibroblasts and myofibroblasts, marked nuclear enlargement, large nucleoli, no mitoses. The features of ANP under electron microscopy are as follows: (i) typical fibroblasts and myofibroblasts with indistinct nuclear fibrous lamina, endothelial cells (ii) amorphous extracellular matrix (fibrin, plasma, cellular debris). 

In summation, the characteristic pathological features of ANP are protracted vascular growth, collection of extracellular amorphous eosinophilic substance, and atypical stromal cells [2, 4]. In our study, from the forth case, we conclude that the angiomatous polyps can also present with facial deformity and bone destruction.

## Conclusions

Angiomatous polyps are rare lesions which entail additional concerns and efforts for diagnosis and surgical intervention. Angiomatous nasal polyps are rapidly developing and aggressively expanding lesions that may turn malignant prior to surgical management. The clinical and imaging features of these lesions are not specific hence they usually boost the consideration of multiple neoplastic lesions in the differential diagnosis. 
